# Acceptance, drivers, and barriers to use eHealth interventions in patients with post-COVID-19 syndrome for management of post-COVID-19 symptoms: a cross-sectional study

**DOI:** 10.1177/17562864231175730

**Published:** 2023-05-27

**Authors:** Julia Schröder, Alexander Bäuerle, Lisa Maria Jahre, Eva-Maria Skoda, Mark Stettner, Christoph Kleinschnitz, Martin Teufel, Hannah Dinse

**Affiliations:** Clinic for Psychosomatic Medicine and Psychotherapy, LVR-University Hospital Essen, University of Duisburg-Essen, Essen, Germany; Center for Translational Neuro- and Behavioral Sciences (C-TNBS), University of Duisburg-Essen, Essen, Germany; Clinic for Psychosomatic Medicine and Psychotherapy, LVR-University Hospital Essen, University of Duisburg-Essen, 45147 Essen, Germany; Center for Translational Neuro- and Behavioral Sciences (C-TNBS), University of Duisburg-Essen, Essen, Germany; Clinic for Psychosomatic Medicine and Psychotherapy, LVR-University Hospital Essen, University of Duisburg-Essen, Essen, Germany; Center for Translational Neuro- and Behavioral Sciences (C-TNBS), University of Duisburg-Essen, Essen, Germany; Clinic for Psychosomatic Medicine and Psychotherapy, LVR-University Hospital Essen, University of Duisburg-Essen, Essen, Germany; Center for Translational Neuro- and Behavioral Sciences (C-TNBS), University of Duisburg-Essen, Essen, Germany; Department of Neurology and Center for Translational Neuro- and Behavioral Sciences (C-TNBS), University Hospital Essen, Essen, Germany; Department of Neurology and Center for Translational Neuro- and Behavioral Sciences (C-TNBS), University Hospital Essen, Essen, Germany; Clinic for Psychosomatic Medicine and Psychotherapy, LVR-University Hospital Essen, University of Duisburg-Essen, Essen, Germany; Center for Translational Neuro- and Behavioral Sciences (C-TNBS), University of Duisburg-Essen, Essen, Germany; Clinic for Psychosomatic Medicine and Psychotherapy, LVR-University Hospital Essen, University of Duisburg-Essen, Essen, Germany; Center for Translational Neuro- and Behavioral Sciences (C-TNBS), University of Duisburg-Essen, Essen, Germany

**Keywords:** acceptance, eHealth, long COVID-19, online interventions, post-COVID-19 syndrome, symptom management, Unified Theory of Acceptance and Use of Technology, UTAUT

## Abstract

**Background:**

Post-COVID-19 syndrome is a new and debilitating disease without adequate treatment options. eHealth could be a reasonable approach for symptom management.

**Objectives:**

This study aims to evaluate the acceptance for eHealth interventions for symptom management in individuals with post-COVID-19 syndrome, as well as drivers and barriers influencing acceptance.

**Design:**

Cross-sectional study.

**Methods:**

This study was conducted from January 19 until 24 May 2022. Recruitment took place with a web-based survey. Acceptance and predictors of eHealth interventions were measured by the extended UTAUT model. Included in the model were the core predictor performance expectancy, social influence, and effort expectancy. Previously diagnosed mental illness was estimated and mental health by using the well-established Generalized Anxiety Disorder Scale-7 and the Patient Health Questionnaire Depression Scale. The effect of sociodemographic and medical data was assessed. Multiple hierarchical regression analyses as well as group comparisons were performed.

**Results:**

342 individuals with post-COVID-19 syndrome were examined. The acceptance of eHealth interventions for symptom management was moderate to high (M = 3.60, SD = 0.89). Acceptance was significantly higher in individuals with lower/other education, patients with moderate to severe symptoms during initial COVID-19 infection, still significantly impaired patients, and individuals with a mental illness. Identified predictors of acceptance were age (β = .24, *p* < .001), current condition including moderate (β = .49, *p* = .002) and still significantly impaired (β = .67, *p* < .001), digital confidence (β = .19, *p* < .001), effort expectancy (β = .26, *p* < .001), performance expectancy (β = .33, *p* < .001), and social influence (β = .26, *p* < .001).

**Conclusion:**

Patients with post-COVID-19 syndrome reported a satisfying level of acceptance and drivers and barriers could be identified. These factors need to be considered for the implementation and future use of eHealth interventions.

## Introduction

The coronavirus disease (COVID-19), caused by the SARS-CoV-2, first emerged in late December 2019.^1^ It has become the causative agent of a severe global pandemic, as declared by the World Health Organization in March 2020.^1,2^ The pandemic led to social distancing and several lockdowns, all to limit the infection with the virus as much as possible.^2^ Despite these actions, the virus spread quickly around the world and infected about 644 million people and caused around 6.6 million deaths (until 11 December 2022).^3^ The most common acute symptoms are fever or chills, cough, fatigue, headache, loss of taste or smell and shortness of breath.^4^ The acute infection most often causes symptoms after 4 to 5 days and up to 4 weeks.^5–7^ In addition, several studies have shown that the pandemic and the post-pandemic period result in increased psychological and mental health problems, including anxiety, chronic stress, and depressions.^8,9^

In addition to the acute infections, there are other conditions caused by COVID-19, such as ‘post-COVID-19 syndrome’,^5–7^ in which symptoms can persist longer than 12 weeks after the initial infection and cannot be explained by an alternative diagnosis.^6,7^ Another stage called ‘ongoing symptomatic COVID-19’ describes the stage between the acute COVID-19 and the post-COVID-19 syndrome in which symptoms occur from four up to 12 weeks.^7^ These stages can be accompanied by different types of symptoms, meaning that in one state new symptoms can occur, whereas others recede or fluctuate.^5,7^ Besides, expression varies in character and severity irrespective of the severity and symptoms of the acute infection.^6,10^ Post-COVID-19 syndrome can affect different organ systems such as cardiological, neuropsychological, pneumological, and neurological, leading to the most common problems including fatigue, reduced quality of life, lower respiratory system problems like cough and dyspnea, chest pain, joint pain, myalgia, concentration issues, and headaches.^5,11,12^

Due to this new infection and the large number of affected people, treatment and support for patients is much needed. The treatment is dependent on patients’ comorbidities, health status including mental health, age, gender, progress of post-COVID-19, and affected organ systems.^13–15^ This complexity highlights the demand for an approach that is interdisciplinary and individualized based on patients’ needs.^13^ Simultaneously, the diverse course of this illness makes diagnosing post-COVID-19 syndrome and developing an individual treatment plan difficult.

Moreover, the pandemic exhausted healthcare workers and overloaded health care centers including limited inpatient and outpatient treatment capacity.^16,17^ Particularly long-term treatment and persistent care needed for post-COVID-19 patients are a significant challenge under these circumstances.^16,17^ These aspects amplify the immense social, economic, and health burden and result in an increased demand for new types of treatments.^13^

eHealth interventions supporting the management of post-COVID-19-related symptoms can provide an effective way to deal with the limitations of the healthcare system and to disburden it in times of pandemic strain.^18^ These interventions offer several benefits to patients, such as addressing questions, coordinating treatment of acute and persistent cases, and reducing the exposure to the virus in case of debilitated or nervous patients. eHealth interventions can be offered anonymously, contact-free, cost-efficient, and are easily accessible.^18–20^ There are many ways to incorporate them into daily life, like smartphone apps, video calls, and electronic messaging.^18^ Nevertheless, downsides of eHealth are concerns about data safety and anonymity, limitations in accessibility, and negative treatment expectations.^21^ In Germany, the Federal Government decided that medical health apps can be prescribed by physicians and costs need to be covered by the statutory health insurance. These conditions are formulated and released as ‘Digital Healthcare Act’ making the usage of digital health apps now more accessible than ever.^22^ However, the implementation of eHealth, especially in Germany, is still in an early stage.

Meanwhile, a meta-analysis has shown equivalent treatment effects of eHealth interventions compared with traditional treatment regarding psychological and somatic disorders.^23^ Even though in most of the studies examining online interventions acceptance was satisfactory,^24^ it is important to further analyze the acceptance and its underlying factors in different patient groups.^21^ Therefore, it is crucial to investigate the individual needs, wishes, and demands toward eHealth by assessing potential drivers and barriers. This is especially relevant for individuals with post-COVID-19 syndrome since there is insufficient research addressing this disease.

The Unified Theory of Acceptance and Use of Technology (UTAUT) is a model to analyze the factors that influence acceptance of new technologies^25^ and the underlying factors of acceptance in telemedicine, respectively, eHealth interventions.^24^ The model has been applied in patients with diabetes, patients with obesity, patients with chronic pain, and aftercare in inpatients and relapse prevention.^19,20,21,26,27^ The UTAUT consist of four core predictors including performance expectancy (PE), effort expectancy (EE), social influence (SI), and facilitating condition (FC).^20,25^ Acceptance is operationalized as behavioral intention (BI) and is predicted by the first three core predictors.^24,25^ PE stands for the extent of individual believes that using the technology will benefit them and increase performance. EE contains the degree of ease while using the intervention. SI describes the importance of individual believes that their social environment approves and believes in the usage of the technology.^25^ BI will lead to usage behavior, which describes the actual usage of the technology (e.g. eHealth interventions).^24^ Actual usage behavior is further predicted by the FC. FC stands for the degree to which the organizational and technical infrastructure is available to the individual for the effective usage of the intervention.^25^

## Objectives

To support a successful implementation of eHealth interventions targeting individuals with post-COVID-19 syndrome, the primary aim of the study was to investigate acceptance regarding eHealth interventions to manage the post-COVID-19 symptoms and factors positively influencing acceptance, as well as potential barriers. Acceptance of eHealth interventions can depend on different sociodemographic factors such as education, age, and mental health status. Further, depression and chronic stress, which are especially important during times of the COVID-19 pandemic, might influence acceptance.^19,20,21^ For this reason, sociodemographic and medical variables including age, education, and mental health, were added as direct predictors to the original UTAUT model. Therefore, another objective was to differentiate between the original model with three predictors, PE, EE, and SI, and the extended UTAUT model with additional predictors.

The research questions of this study are listed below.

1. To what extent do patients with post-COVID-19 syndrome accept eHealth interventions for symptom management?
2. How does the acceptance differ between patients with different sociodemographic and medical characteristics?
3. What are potential drivers of and barriers to acceptance?
4. Is the described extended UTAUT model preferable to the original UTAUT model?

## Materials and methods

### Study design and participants

A cross-sectional survey-based study was conducted to assess acceptance, drivers, and barriers of eHealth interventions to manage post-COVID symptoms. No intervention was offered. The participants of this study were recruited with flyers in different hospitals (Essen University Hospital, Klinikum Osnabrück, Schüchtermann-Klinik Bad Rothenfelde), different rehabilitation clinics for post-COVID-19 patients (e.g. MEDIAN clinics, Nordseeklinik Westfalen), and (online) self-help group communities (e.g. Long Covid patient advocacy group Bochum, Post-COVID patient advocacy group Munich, Post-COVID patient advocacy group Tübingen). Patients were recruited between 19 January and 24 May 2022. Inclusion criteria were age of 18 years or higher, good German language skills, internet access, history of a confirmed COVID-19 infection and current post-COVID-19 symptoms. The post-COVID-19 symptoms were assessed according to the clinical case definition by the WHO.^5^ The survey was offered via the online platform Unipark and participation was anonymous, voluntary, and without monetary compensation. The participants had to accept an electronic informed consent form before starting the assessment.

**Figure 1.**
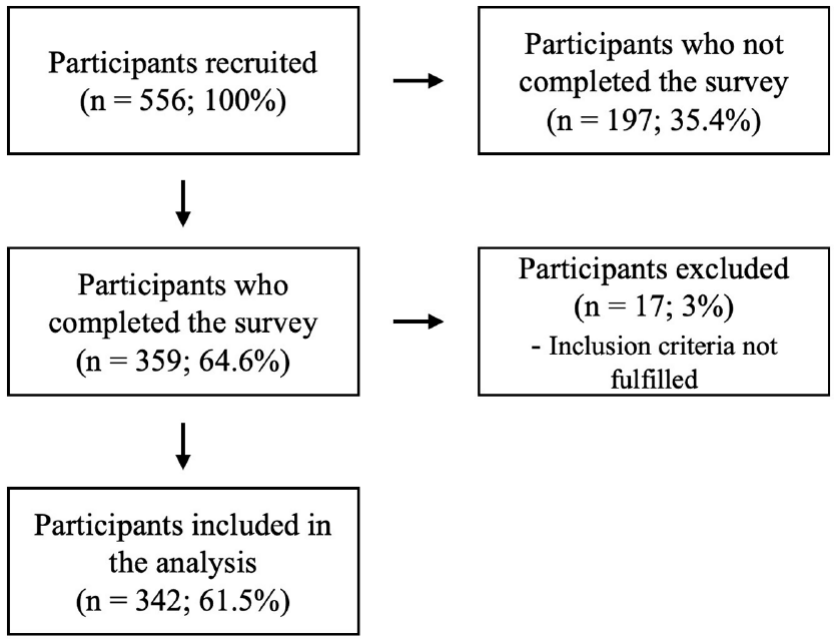
Overview of the participants’ flow.

Of *N* = 556 participants who initially started the survey, *N* = 359 completed the survey resulting in a completion rate of 64.6%. *N* = 17 participants were excluded because the inclusion criteria were not fulfilled. Therefore, *N* = 342 participants were included in the final data analysis. See Figure 1 for an overview.

The average time the participants needed to complete the survey was 14 min. The study was executed in accordance with the Declaration of Helsinki and was approved by the Ethics Committee of the Medical Faculty of the University of Duisburg-Essen (19-89-47-BO).

### Assessment instruments

The assessment was divided into eight modules and contained sociodemographic, medical, and mental health questions. In addition, a modified UTAUT model was used to assess the acceptance of eHealth interventions to manage symptoms and self-generated items were used to examine eHealth-related data. All self-generated items and scales regarding eHealth were previously used and well-established.^19,26^

Participants were asked to rate three items regarding their digital confidence on a 5-point Likert-type scale (e.g. ‘How confident are you in using digital media?’, 1 = very insecure to 5 = very confident). Internal consistency of this scale was excellent, with Cronbach’s α = .95. Further, digital overload was assessed with three items and answers were given on a 5-point Likert-type scale (e.g. ‘I feel burdened by the constant availability via cell phone or e-mail’. 1 = does not apply to 5 = does fully apply). Cronbach’s α in this study was .72 for digital overload, indicating acceptable consistency.

To assess the acceptance of eHealth interventions for symptom management and its predictors in post-COVID-19 patients, a modified version of the UTAUT model^25^ was used. Responses were given on a 5-point Likert-type scale, with answers ranging from 1 = totally disagree to 5 = totally agree. To assess acceptance, operationalized as BI, and its underlying core predictors (PE, SI, EE), three items per construct were used. In this study, Cronbach’s α was .89 for acceptance (BI), .89 for PE, .81 for SI, and .71 for EE, which indicated sufficient to high internal consistency.

Regarding mental health, patients were asked if they had been diagnosed with mental illness. Depressive symptoms were screened with the Patient Health Questionnaire-8 (PHQ-8), which consists of eight items. Responses are given on a 4-point Likert-type scale, ranging from 0 = not at all to 3 = nearly every day. A score of or above 10 indicates major depression symptoms.^28^ Cronbach’s α in this study was .83, indicating high internal consistency. Generalized Anxiety Disorder 7 (GAD-7) was used to examine generalized anxiety symptoms. The scale consists of seven items and is rated on a 4-point Likert-type scale, ranging from 0 = not at all and 3 = nearly every day. Cut-off score of ⩾ 5, ⩾ 10 and ⩾ 15 indicates mild, moderate, and severe generalized anxiety symptoms, respectively. Internal consistency was high, with Cronbach’s α of .90.

To evaluate the post-COVID-19 syndrome, current symptoms were assessed. In addition, several anamnestic details including the infection date, type of treatment (hospital stay, intensive care), variety of symptoms (e.g. cough, fever, other), severity of symptoms of COVID-19 infection, and current condition were assessed.

Finally, sociodemographic data were collected. Sociodemographic data included age, gender, marital status, educational level, occupational status, and place of residence (population size).

### Statistical analysis

Statistical analyses were conducted using SPSS Statistics version 26 (IBM, New York, NY, USA) and the software R (4.0.3). First, sum scores for GAD-7 and PHQ-8, as well as mean scores for the UTAUT model (BI, PE, EE, SI), were calculated. In addition, mean scores and standard deviations for self-generated items were calculated. According to previous research,^20,21^ acceptance (= BI) scores were split into three ranges: Low acceptance was determined by scores between 1 and 2.34, moderate acceptance between 2.35 and 3.67, and high acceptance between 3.68 and 5. Differences in acceptance were examined for educational level, progression of COVID-19, current condition, and mental illness. Analyses of variance (ANOVAs) with post hoc tests and independent *t* tests were used for this. Bonferroni correction was applied to adjust *p* values for multiple comparisons. Levene’s test was used to test for homoscedasticity. A normal distribution of residuals was assumed due to the sample size. Multiple hierarchical regression analysis was applied to investigate possible predictors of acceptance. The following predictors were included block-wise: (1) sociodemographic data, (2) medical and psychometric data, (3) eHealth variables, and (4) UTAUT predictors (PE, EE, SI). No multicollinearity could be detected since variance inflation factor (VIF) values for testing multicollinearity were all VIF ⩽ 2.^29^ The q–q plots of the residuals were visually inspected and showed no signs of violations against normality. Accordingly, normal distribution of the residuals can be assumed. Homoscedasticity was proven based on a scatter plot of the standardized residuals and the adjusted predicted values. Finally, the restricted UTAUT model, only including PE, EE, and SI as core predictors, was compared with the extended UTAUT model using an ANOVA. The level of significance was set to α < .05 for all tests. Effect sizes are reported and interpreted according to Cohen (1988), with values around 0.2, 0.5, and 0.8 being considered as small, medium-sized, and large effect, retrospectively.^30^

## Results

### Study population

The mean age of this sample of individuals with post-COVID-19 syndrome was M = 36.74 (SD = 12.84) years. The youngest participant was 18 years old, and the oldest was 70 years old. Individuals with post-COVID-19 syndrome showed high digital confidence (M = 4.09, SD = 1.05, range 1–5). Experienced digital overload was low in this sample (M = 2.74, SD = 0.91, range 1–5). See Table 1 for a detailed overview of the sociodemographic data.

**Table 1.** Sociodemographic data.

	*n* (%)
Gender	
Male	66 (19.3)
Female	274 (80.1)
Diverse	2 (0.6)
Marital status	
Single	99 (28.9)
In a relationship	114 (33.3)
Married	112 (32.7)
Divorced/separated/widowed	12 (3.5)
Other	5 (1.5)
Educational status	
(Lower) secondary education/other	85 (24.8)
Higher education entrance qualification	102 (29.8)
University education	155 (45.4)
Occupational status	
In education	84 (24.6)
Unemployed	16 (4.7)
Sick leave	96 (28.1)
Partially employed	51 (14.9)
Fully employed	70 (20.5)
Retired	5 (1.5)
Other	20 (5.8)
Place of residence (population size)	
Large city (>100,000 residents)	151 (44.2)
Medium-sized city (>20,000 residents)	81 (23.7)
Small town (>5,000 residents)	52 (15.2)
Rural area (>5,000 residents)	58 (17.0)
Total	342 (100.0)

In this sample, 23.7% (*n* = 81) were currently affected by a mental illness. The most common persisting symptom of COVID-19 was headache or pain in limbs (59.1%; *n* = 202), followed by cough (36.0%; *n* = 123) and shortness of breath (35.1%; *n* = 120). 56.4% (*n* = 193) of the participants reported additional symptoms, such as fatigue, memory problems, difficulty concentrating, nerve and muscle pain, hair loss, tachycardia, and dizziness. For an overview of further medical and psychometric data, see Table 2.

**Table 2.** Medical and psychometric data.

	M (SD)	*n* (%)
COVID-19 progression (%)		
No to mild symptoms		160 (50.0)
Moderate to severe symptoms		160 (50.0)
Hospital treatment for COVID-19 infection		22 (5.6)
Treatment in intensive care for COVID-19 infection		8 (2.1)
Current condition		
Good		62 (18.1)
Moderate		108 (31.6)
Still significantly impaired		172 (50.3)
GAD-7	7.39 (5.12)	
No to low anxiety symptoms (<5)		119 (34.8)
Mild anxiety symptoms (<10)		117 (34.2)
Moderate anxiety symptoms (<15)		71 (20.8)
Severe anxiety symptoms (⩾15)		35 (10.2)
PHQ-8	9.99 (5.31)	
No depressive symptoms (<10)		167 (48.8)
Depressive symptoms (⩾10)		175 (51.2)
Total		342 (100.0)

GAD-7, Generalized Anxiety Disorder Scale-7; PHQ-8, Patient Health Questionnaire Depression Scale; SD, standard deviation.

### Acceptance by sociodemographic and medical data

The overall acceptance was moderate to high (M = 3.60, SD = 0.89). 10.2% (*n* = 35) of the participants showed low acceptance, 38.0% (*n* = 130) reported moderate acceptance and over half of the participants (51.8%; *n* = 177) reported high acceptance.

An ANOVA revealed significant differences in acceptance between levels of education (*F*(2, 339) = 5.40, *p*_adj_ = .020, η² = .03). Tukey post hoc analysis showed that individuals with (lower) secondary education or other education reported significantly higher acceptance than individuals holding an academic degree (*p*_adj_ = .003).

Individuals with no to mild symptoms during the initial COVID-19 infection reported a significant lower acceptance than individuals with moderate to severe symptoms (*t*(318) = -3.28, *p*_adj_ = .005, *d* = .37).

An ANOVA revealed significant differences between different levels of current condition (*F*(2, 339) = 22.83, *p*_adj_ < .001, η² = .12). Tukey post hoc analysis found that participants in a good condition showed the lowest acceptance, followed by participants in a moderate condition and that the highest acceptance was reported by individuals who were still significantly impaired (all *p*_adj_ ⩽ .007).

Acceptance of eHealth interventions was significantly higher in participants with mental illness compared with individuals without mental illness (*t*(340) = 3.46, *p*_adj_ = .002, *d* = .44).

### Predictors of acceptance

To perform multiple hierarchical regression analysis, data from *n* = 23 participants had to be excluded. Two subjects had reported their gender as diverse and for 21 participants data for relevant predictors were missing.

Multiple hierarchical analysis revealed that the sociodemographic predictors included in the first step explained 8.5% of the variance of acceptance (*R*^2^ = .085, *R*^2^_adj_ = .064, *F*(7,311) = 4.12, *p* < .001). In the first step, acceptance was significantly predicted by *Age* (β = .24, *p* < .001).

The second step included psychometric and medical data as predictors (*R*^2^ = .174, *R*^2^_adj_ = .142, *F*(12,306) = 5.38, *p* < .001), and the explained variance increased significantly to 17.4% (∆*R*^2^ = .089, *F*(5,306) = 14.63, *p* < .001). In this step, additional significant predictors of acceptance were *Current condition: Moderate* (β = .49, *p* = .002) and *Still significantly impaired* (β = .67, *p* < .001).

eHealth-related predictors, which were included in the third step (*R*^2^ = .209, *R*^2^_*adj*_ = .173, *F*(14,304) = 5.74, *p* < .001), increased the explained variance significantly to 20.9% (∆*R*^2^ = .035, *F*(2,304) = 14.23, *p* < .001). *Digital confidence* was another significant predictor of acceptance in the third step (β = .19, *p* < .001).

The final step included the UTAUT predictors (*R*^2^ = .632, *R*^2^_adj_ = .612, *F*(17,301) = 30.45, *p* < .001) and the explained variance was 63.2% (∆*R*^2^ = .423, *F*(3,301) = 115.51, *p* < .001). *Effort expectancy* (β = .26), *Performance expectancy* (β = .33), and *Social influence* (β = .26) were significant predictors of acceptance (*p* < .001). Table 3 gives an overview of the hierarchical regression model.

**Table 3.** Hierarchical regression model of acceptance.

Predictors	*B*	β	*T*	*R* ^2^	∆*R*^2^	*p*
(Intercept)	−.53	−.44	−1.84			.067
Step 1: sociodemographic data				.085	.085	
Age	.01	.12	2.73			.007
Gender: female	.14	.16	1.76			.079
Place of residence: medium-sized city	−.04	−.05	−0.53			.595
Place of residence: small town	.02	.02	0.19			.846
Place of residence: rural area	−.13	−.15	−1.45			.148
Education: higher education entrance qualification	.08	.09	0.91			.365
Education: university education	.03	.03	0.40			.689
Step 2: medical and psychometric data				.174	.089	
COVID-19 progression: moderate to severe symptoms	.09	.10	1.26			.208
Current condition: moderate	.26	.30	2.72			.007
Current condition: still significantly impaired	.35	.39	3.22			.001
Mental illness: no	−.03	−.04	−0.43			.671
PHQ-8 sumscore	.00	.02	0.43			.670
Step 3: eHealth variables				.209	.035	
Digital confidence	.05	.06	1.59			.113
Digital overload	−.01	−.01	−0.28			.778
Step 4: UTAUT predictors				.632	.423	
Performance expectancy	.34	.33	7.43			<.001
Effort expectancy	.29	.26	5.81			<.001
Social influence	.29	.26	6.02			<.001

∆*R*^2^, changes in *R*^2^; *B*, unstandardized beta; GAD-7, Generalized Anxiety Disorder Scale-7; PHQ-8, Patient Health Questionnaire Depression Scale; *R*², determination coefficient; *t*, test statistic; UTAUT, Unified Theory of Acceptance and Use of Technology; β, standardized beta.

*N* = 319. In Steps 2, 3, and 4, only the newly included variables are presented.

### Comparison between original UTAUT and extended UTAUT model

In the final step, the extended UTAUT model (*R*^2^ = .632, *R*^2^_adj_ = .612) was compared with the original UTAUT model, which only contained the three core predictors PE, EE, and SI (*R*^2^ = .560, *R*^2^_adj_ = .556). The extended UTAUT model showed a significantly higher explained variance than the original UTAUT model (*F*(14,301) = 4.21, *p* < .001), which indicates that the UTAUT model of acceptance could be improved by adding additional variables.

## Discussion

The aim of this study was to assess the acceptance toward eHealth interventions to manage symptoms as well as the influencing factors in patients with post-COVID-19 syndrome. Since there are no effective treatment options so far, these findings could have an important impact on the implementation of eHealth interventions to assist affected patients. The overall acceptance was moderate to high. Over half of the participants reported high acceptance. Individuals with no to mild symptoms during the initial infection showed lower acceptance in comparison to individuals with moderate to severe symptoms. Besides, individuals who are still significantly impaired reported higher acceptance than patients in a currently good health. Further, individuals with (lower) secondary education and patients with a mental illness demonstrated higher acceptance.

In addition, different factors including age, current condition, digital confidence, and the three UTAUT predictors EE, PE, SI were significant predictors influencing acceptance toward eHealth interventions for post-COVID-19 syndrome. The comparison of the original and extended version of the UTAUT model describes that the extended version shows higher explained variance. All these factors should be considered for further improvement and sustained implementation of eHealth interventions.

Results of this study highlighted that acceptance of eHealth interventions was significantly higher in patients who are more psychologically burdened. Mental illness appears to be significantly associated with an increased acceptance which supports several other studies describing the same phenomena.^19,21,31^ Since mental illness and post-COVID-19 symptoms seemed to be connected,^14,15^ an interdisciplinary treatment approach is necessary^13^ in which eHealth interventions could be an important therapy option for symptom management.

In addition, patients who are still moderately to significantly impaired after COVID-19 infection expressed higher acceptance than patients in currently good condition. This is in line with the results of Stoppok *et al.*^27^ showing higher acceptance in more burdened individuals affected by chronic pain. In addition, patients with moderate to severe symptoms during the initial COVID-19 infection also showed higher acceptance. Furthermore, this combination of initially stronger symptoms and still significant impairment could lead to additional concerns, and therefore those affected could be open to accept new eHealth interventions. Besides, patients with this combination often previously tested more treatment and coping options. Consequently, they could be more willing to continue testing new interventions looking for helpful ways treating and managing their symptoms and disease.^27^ The high acceptance in this group of individuals showing openness and interest in eHealth interventions is useful for further developments since to date there are only a limited number of treatment options for post-COVID-19 syndrome. Precisely, these individuals could use treatment options as eHealth since usage is not restricted by physical weakness or problems in coping with everyday life.

An important sociodemographic predictor in this study was age. Age as a predictor is concordant with previous studies investigating acceptance toward eHealth interventions.^19,20,21,26,27^ Young people often use digital media naturally because they grew up with the internet.^32^ Increasing acceptance in older people could be achieved by addressing older people specifically by, for example, face-to-face introductory courses, video explanations describing how to use eHealth interventions, or tailored design aspects (e.g. bigger font size).

Unlike other studies,^27,33^ our results showed differences in acceptance toward eHealth interventions dependent on place of residence. It could be assumed that since there is a general lack of treatment options for post-COVID-19 syndrome due to novelty and complexity of the disease,^13^ patient care for COVID-19 symptoms does not differ depending on place of residence. This further shows that the wish for treatment options like eHealth interventions has no difference dependent on the place of residence.

Higher digital confidence is associated with higher acceptance of eHealth interventions. The high level of digital confidence in this study could be associated with higher familiarity and tolerance of online media in general. This could explain the increased acceptance toward medical online interventions. Several studies show that Internet anxiety often negatively impacts acceptance.^24,26,34^ In addition, other studies show similar positive connections between digital confidence and acceptance.^19,27,35^ Digital confidence is, therefore, a relevant intervention factor to increase acceptance and actual usage.

The core predictors SI, EE, and PE explained 63.2% of the variance in acceptance and are significant predictors in the extended UTAUT model for acceptance of eHealth interventions. This relationship was also observed in studies analyzing individuals with obesity and overweight, diabetes, and chronic pain.^19,21,26,27^ Furthermore, PE was found to be the predictor with the highest association with acceptance,^19,20,21^ which highlights that the acceptance is dependent on the beliefs of post-COVID-19 patients that the interventions could help them. Besides, it could be shown that the original UTAUT model is a reasonable way to predict the acceptance toward eHealth interventions for symptom management in post-COVID-19 patients. In addition, the comparison between the original and the extended UTAUT model indicated that the extended version, which includes additional predictors, is able to explain a higher level of variance of acceptance. This result has been previously described in other studies,^19,27^ whereas another study reported opposite results.^26^ For the implementation of eHealth interventions, other factors besides the core predictors, including medical and sociodemographic data, should be considered. Finally, the UTAUT model is an evidence-based foundation for the implementation of eHealth interventions for post-COVID-19 patients and should be used for further development of these interventions.

To conclude, this study shows that different factors, including sociodemographic and medical data, are associated with the acceptance for eHealth interventions to manage symptoms in post-COVID-19 patients. These findings underline the complexity of the concept of acceptance of eHealth interventions and the challenges for the implementation of innovative eHealth offers. Therefore, the observed predictors of acceptance need to be considered during the development of specialized and need-based eHealth interventions.

## Limitations

The following limitations should be considered when interpreting the results of this study. The assessment was only available online. Since access to the Internet is not equally distributed between age groups, we can assume that the digital safety and usage as well as the interest in using digital health interventions may be higher among this group of individuals.^36^ This is also shown since the study sample was relatively young. Therefore, selection bias cannot be ruled out. In addition, participants required a confirmed COVID-19 infection in the past and ongoing post-COVID-19 symptoms to be suitable participants for the survey. Since the post-COVID-19 diagnosis could not be objectively confirmed, self-report bias cannot be ruled out. Furthermore, this study focused on analyzing the acceptance of the interventions to manage symptoms and not further the actual usage behavior. In the UTAUT model^25^ BI is associated with actual use behavior. However, it is not clear if the BI to use the intervention can be equally described as the actual use behavior. This is described as the intention–behavior gap,^37^ which describes that the intention to do something does not lead to the use behavior. Nevertheless, future studies should further focus on the actual usage behavior for assessing the acceptance rather than the BI to avoid the intention–behavior gap. Finally, it should be considered that additional factors not analyzed in this study could be also significant predictors for variance of acceptance in post-COVID-19 patients.

## Conclusion

This study suggests that the acceptance toward eHealth interventions to manage symptoms was moderate to high among post-COVID-19 patients. PE, EE, and SI have been proven as predictors of acceptance. Furthermore, the results have shown that there are additional predictors including age, current condition, and digital confidence. Until now, there are no effective treatment options to cure post-COVID-19 syndrome. eHealth interventions could be a reasonable way to improve and manage symptoms in post-COVID-19 patients. The results of this study aim to support the implementation and sustained usage of such eHealth interventions.

## Declarations

**Ethics approval and consent to participate:** The study was executed in accordance with the Declaration of Helsinki and was approved by the Ethics Committee of the Medical Faculty of the University of Duisburg-Essen (19-89-47-BO). All participants had to accept an electronic informed consent form before starting the assessment.

**Consent for publication:** Not applicable.

**Availability of data and materials:** The data supporting the presented findings are available upon reasonable request to the corresponding author.
